# Efficient methods for acute stress detection using heart rate variability data from Ambient Assisted Living sensors

**DOI:** 10.1186/s12938-021-00911-6

**Published:** 2021-07-29

**Authors:** Benedek Szakonyi, István Vassányi, Edit Schumacher, István Kósa

**Affiliations:** 1 Medical Informatics Research & Development Center, University of Pannonia, Egyetem u. 10, 8200 Veszprém, Hungary; 2 Cardiac Rehabilitation Institute of the Military Hospital, Balatonfüred, Hungary; 3 Department of Preventive Medicine, University of Szeged, Szeged, Hungary

**Keywords:** Ambient Assisted Living, Stress detection, Heart rate variability, Wearable sensor, Stroop colour word test, Trier social stress test, State–Trait Anxiety Inventory

## Abstract

**Background:**

Using Ambient Assisted Living sensors to detect acute stress could help people mitigate the harmful effects of everyday stressful situations. This would help both the healthy and those affected more by sudden stressors, e.g., people with diabetes or heart conditions. The study aimed to develop a method for providing reliable stress detection based on heart rate variability features extracted from portable devices.

**Methods:**

Features extracted from portable electrocardiogram sensor recordings were used for training various classification algorithms for stress detection purposes. Data were recorded in a clinical trial with 7 participants and two stressors, the Trier Social Stress Test and the Stroop colour word test, both validated by standardised questionnaires. Different heart rate variability feature sets (all, time-domain and non-linear only, frequency-domain only) were tested to investigate how classification performance is affected, in addition to various time window length setups and participant-wise training sessions. The accuracy and F1 score of the trained models were compared and analysed.

**Results:**

The best results were achieved with models using time-domain and non-linear heart rate variability features with 5-min-long overlapping time windows, yielding 96.31% accuracy and 96.26% F1 score. Shorter overlapping windows had slightly lower performance, with 91.62–94.55% accuracy and 91.77–94.55% F1 score ranges. Non-overlapping window configurations were less effective, with both accuracy and F1 score below 88%. For participant-wise learning, average F1 scores of 99.47%, 98.93% and 96.1% were achieved for feature sets using all, time-domain and non-linear, and frequency-domain features, respectively.

**Conclusion:**

The tested stress detector models based on heart rate variability data recorded by a single electrocardiogram sensor performed just as well as those published in the literature working with multiple sensors, or even better. This suggests that once portable devices such as smartwatches provide reliable hear rate variability recordings, efficient stress detection can be achieved without the need for additional physiological measurements.

**Supplementary Information:**

The online version contains supplementary material available at 10.1186/s12938-021-00911-6.

## Background

As stress became one of the main problems of modern societies, its adverse effects are quite well known even to the general population. Whether physical, emotional or mental strain, the prolonged presence of stress contributes to developing chronic diseases such as diabetes, cardiovascular and respiratory conditions, depression and even some forms of cancers [1–6]. Due to these health concerns, there has been an increased effort to develop for the detection and assessment of stressful events in everyday situations to support people in minimising these harmful effects. The presence and level of stress in clinical practice are confirmed by taking and analysing blood or saliva samples to measure the cortisol hormone level [7]. While it is the most precise method for measuring stress, it requires specific lab equipment and medical personnel, making it impractical for everyday usage. This leads to a need for finding alternative methods. Ambient Assisted Living (AAL) applications are such alternatives, as they aim to provide unobtrusive lifestyle support in daily living situations. They achieve this via combining different types of sensors, mobile devices, computers, networks and software solutions to monitor and assist users when needed. AAL stress detection approaches are generally categorised into two main groups: those dealing with chronic stress detection and those aimed at acute stress.

Chronic stress assessment is mainly executed based on data recorded throughout multiple days or weeks, sometimes months. In general, longer time intervals spanning hours are identified and classified as stressful or resting periods, while some solutions also try to recognise physical activity and sleeping phases as well [8–10]. On the other hand, detecting the presence or build-up of acute stress is usually initiated by analysing recordings of a couple of minutes, covering a total of 0.5–1 h at most.

While both acute and chronic stress has a high impact on the quality of life, dealing with acute stress situations facilitates negating chronic stress. Moreover, the short-term, high-intensity effects of acute stress pose additional hazards for some people, e.g., those with cardiovascular conditions (increased heart rate and blood pressure) [2, 11] or diabetes (rapid blood glucose level changes) [12]. Therefore, as AAL solutions advance, the interest in the research community for acute stress detection increases.

Several different AAL sensor types and solutions have been proposed and assessed in the stress detection literature. Some of these solutions work by using just one selected sensor type, while others simultaneously record data from multiple sources. Single-sensor-based solutions often use electrocardiogram (ECG) [13–22] or photoplethysmogram (PPG) [23–26] signals, usually to obtain heart rate variability (HRV) features. In other cases, electrodermal activity (EDA) [27] or electromagnetic waves ("bioradar") [28] are used. Additional sensors used by multimodal approaches include the galvanic skin response (GSR) [29–32], respiration [29, 30, 33], electromyography (EMG) [34], and even such data as physical (in)activity, calories used or sleep quality, measured by activity trackers [9, 10]. The focus of the research is shifting to developing methods that utilise compact, inexpensive wearable sensor devices suitable for everyday use for both approaches. Such devices are chest belts [13, 22, 33] or ECG-infused clothing [19], wrist bracelets or activity trackers [9, 10, 20, 27], or other portable ECG devices [13, 21]. Unfortunately, these are not yet without some drawbacks. Their main problem is that while most provide some sort of averaged pulse data, HRV feature extraction requires more precise, pulse-to-pulse measurements at millisecond precision for reliable stress detection. Regarding battery lives in general, progress has allowed once-a-week charging, but there is still room for improvements.

While using multiple different modalities can yield better results as more data are recorded, it also increases both computational and system complexity, costs and operational resource (energy) needs. For this reason, our study uses a single, portable HRV sensor.

**Fig. 1:**
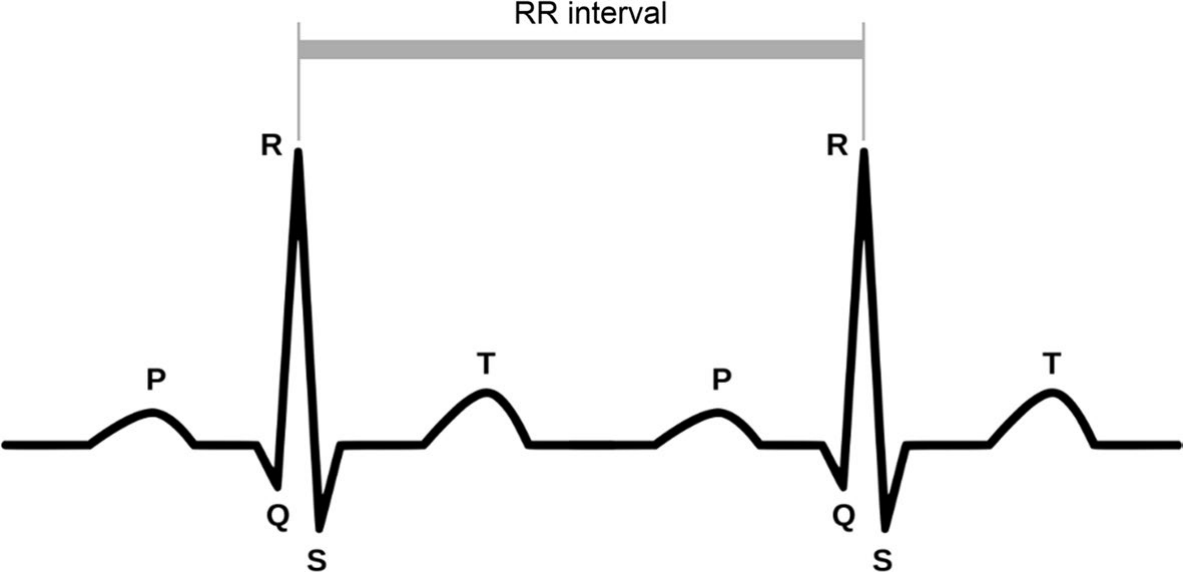
The schematic representation of the RR interval

HRV features describe the fluctuations present in the length of successive heartbeat intervals, and are known to be impacted by stress [35]. The distance between two successive heartbeats, i.e. the distance between the R wave of their QRS complexes, is called the RR interval (as illustrated in Fig. 1).

### State of the art

Table 1 presents some of the most relevant studies published recently in the field of acute stress detection.

**Table 1:** Recent and relevant studies on acute stress detection

Paper	Subjects (number, age)	Sensors	Stressing procedure	Classification methods	Window length (s)	Results
Ham 2017 [23]	6, 28.67 ± 3.3y	PPG (HRV)	Arithmetic stress, environmental stress, both while in Virtual Reality	LDA	240 s, non-overlapping	74% (baseline), 81% (mild stress), 82% (severe stress) *Accuracy*
Pereira 2017 [19]	14, 20-26y	ECG (HRV)	Trier Social Stress Test	N/A	50-220 s, non-overlapping	N/A
Zangróniz 2018 [24]	45, 20-28y	PPG (PRV)	IAPS	Decision Tree	70 s	82,35% *Accuracy*
Lawanont 2019 [10]	10, 20-26y	Activity tracker (FitBit)	Real-life work shifts	KNN, SVM, Decision Tree	1 h	84.1% (decision tree) *Accuracy*
Moridani 2020 [20]	20, 25 ± 5y	ECG (HRV)	Physical activity, arithmetic task, emotional stress (horror movie)	CNN	300 s (40 s for “mini emotional stress”)	97.9% (cognitive), 94.4% (emotional) *F1 score*
Pourmohammadi 2020 [34]	34, 25.4 ± 4.2y	EMG, ECG (HRV)	Arithmetic task, Stroop, environmental stressing	SVM	60 s, non-overlapping	100% (2 levels), 97.6% (3 levels), 96.2% (4 levels) *Accuracy*
Rodriguez-Arce 2020 [29]	21, 18-21y	GSR, PPG, body temp., breathing	Arithmetic task, electrocutaneous stimulation	KNN, SVM	~ 50 s (2500 samples per window)	90% (KNN), 95% (SVM) *Accuracy*
Sánchez-Reolid 2020 [27]	147, 31.4 ± 8.03y (w), 36.3 ± 4.99y (m)	EDA	IAPS	SVM, D-SVM	1-40 s, overlapping and non-overlapping	83% (SVM), 92% (D-SVM) *F1 score*
Zalabarria 2020 [30]	42, 22.88 ± 3.1y	ECG (HR), GSR, breathing	3D puzzle	Fuzzy algorithm	20 s sliding window	91.15% (stress), 96.61% (relax) *F1 score*
Zubair 2020 [26]	14, 25-30y	PPG (HRV)	Arithmetic tasks, Stroop	QDA, SVM	60 s, non-overlapping	94.33% (SVM), 89.73% (QDA) *Accuracy*

*LDA* linear discriminant analysis, *CNN* Convolutional Neural Network, *KNN* K-nearest neighbours, *(D-)SVM* (Deep-)Support Vector Machine, *QDA* quadratic discriminant analysis

Since cortisol-based measurements are infeasible for everyday solutions, and even in most clinical trials, some other “gold standard” measurements are usually required to confirm that stress was successfully induced during a trial. A solution for this problem is using scientifically validated psychological tests. One such well-known and frequently used test is the State–Trait Anxiety Inventory (STAI) [36, 37], a questionnaire used to get self-reported assessments from participants about their perceived stressfulness. Still, there are examples of research done without such validation methods, raising some concerns about the validity of the stressor(s) used (and the data recorded).

There are numerous different methods reported in the stress detection literature for inducing stress. These include different arithmetic tasks [20, 26, 28, 29, 34], games/puzzles [25, 30], exam-like conditions [14, 16, 19, 31], and everyday situations such as driving [38, 39] or work shifts [9, 10, 33]. However, not all of these are standardised and reliable stressors, only ad hoc methods designed and implemented by the researchers themselves, often without psychological expertise. This decreases the reliability of the input data sets, especially for cases where not even golden standard measurements are used to justify the stressor’s effectiveness.

Amongst generally accepted stressing methods are the International Affective Picture System (IAPS) [40] (often used together with the International Affective Digitized Sounds (IADS) [41]), the Socially Evaluated Cold Pressor Test (SECPT) [42], the Trier Social Stress Test [43] and the Stroop colour word test [44]. These stressors are well documented and offer clear and well-detailed script protocols for researchers to ensure good data quality. Not all research aspects can be covered by them, though, leaving room for different trial configurations. For example, such an aspect is the age of the selected participant group.

As shown in Table 1, most recent trials included only relatively young subjects, usually university students (probably as students were available for academic researchers). This point should be improved for two reasons. First, stress-related diseases are known to pose great(er) risks for older adults (people aged 50 and above) [45–47], making them a more important target group for stress support. Thus, observations based solely on younger individuals cannot be expected to match other age groups fully. Second, notable differences in reactions given for stressful situations can be observed even amongst similarly aged people, which can be even more diverse if different generations are compared—not just from a physiological aspect (age-specific bodily functions), but from psychological and sociological aspects (how people were “taught” to react) as well.

### Research objectives and motivation

The main objective of the work presented was to develop a method for stress detection for AAL applications, by using HRV data obtained from a single sensor. The research was designed to use standardised stressing methods (Stroop, Trier tests) and a standardised method for validating that the stressors were implemented properly (STAI questionnaire), an approach missing from many similar studies. Moreover, multiple time window and input set configurations, and different modelling algorithms have been tested to find the best-performing solution.

## Results

### STAI questionnaire and cortisol test results

The State–Trait Anxiety Inventory (STAI) scores received are shown in Table 2. As the scores showed that the Stroop tests have failed to induce stress in several participants, these sessions’ measurements were not used as stressful data in the model building process.

**Table 2:** STAI scores before and after each stressing session

	P1	P2	P3	P4	P5	P6	P7
TRIER Before	39	31	30	28	37	26	35
After	48	43	49	47	66	26	74
STROOP Before	36	34	39	23	44	20	53
After	34	35	38	24	37	20	37

As the sample of four people tested with saliva-cortisol tests is relatively small, no significant conclusions could be drawn. Nevertheless, the results showed that the Trier test caused an increase between 31 and 42% in participants’ cortisol levels, while these values only decreased for the Stroop test sessions (between 2 and 8%).

### Model results

**Fig. 2:**
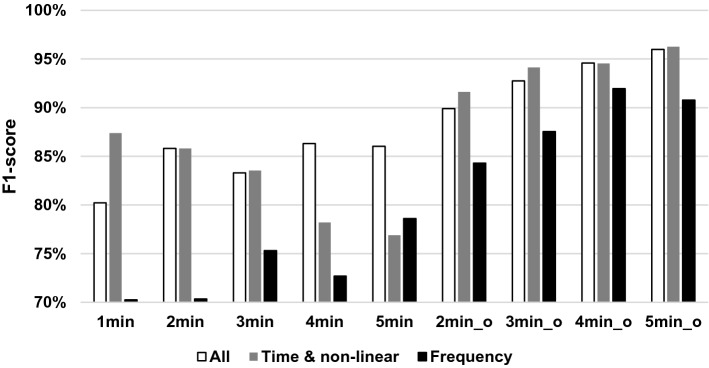
F1 scores for different time windows and the three feature sets of configuration 1 (_o denoting overlapping time window setups)

The F1 scores of the best-performing classifier models for all three HRV feature sets used in configuration 1 and the different time window setups are shown in Fig. 2.

HRV feature set-wise detailed results are given in Tables 3, 4 and 5. The overlapping time windows were found to have better performance in general. The 5-min-long overlapping time window setup yielded the best prediction results for both the all-HRV feature set and the time/non-linear only feature set as well. Using frequency-domain features provided only slightly lower performances than the two other sets. For this set of features, the 4-min-long overlapping time window was found to have the best results.

**Table 3:** Model performances for given time windows, using all HRV features. The best results in boldface

Window length (min)	Non-overlapping windows					Overlapping windows			
	1	2	3	4	5	2	3	4	5
Accuracy	80,70%	86,03%	85,10%	86,67%	85,94%	89,82%	92,94%	94,68%	**96,00%**
Sensitivity	80,51%	84,62%	79,60%	83,89%	86,25%	89,67%	90,88%	93,06%	**95,69%**
Specificity	80,89%	87,44%	90,38%	89,44%	85,63%	90,00%	95,00%	96,29%	**96,31%**
F1 score	80,22%	85,82%	83,30%	86,31%	86,03%	89,91%	92,74%	94,58%	**95,99%**

**Table 4:** Model performances for given time windows, using time-domain and non-linear HRV features

Window length (min)	Non-overlapping windows					Overlapping windows			
	1	2	3	4	5	2	3	4	5
Accuracy	87,53%	86,41%	84,90%	81,11%	78,75%	91,77%	94,19%	94,60%	**96,31%**
Sensitivity	86,58%	84,87%	82,80%	72,22%	74,38%	89,86%	93,24%	93,71%	**95,23%**
Specificity	88,48%	87,95%	86,92%	90,00%	83,13%	93,70%	95,15%	95,48%	**97,38%**
F1 score	87,39%	85,80%	83,54%	78,20%	76,89%	91,62%	94,13%	94,55%	**96,26%**

**Table 5:** Model performances for given time windows, using frequency-domain HRV features only

Window length (min)	Non-overlapping windows					Overlapping windows			
	1	2	3	4	5	2	3	4	5
Accuracy	71,33%	73,33%	77,65%	77,22%	80,00%	84,69%	87,79%	**92,10%**	90,92%
Sensitivity	68,61%	65,90%	73,60%	67,78%	74,38%	82,57%	86,32%	**90,81%**	89,85%
Specificity	74,05%	80,77%	81,54%	86,67%	85,63%	86,85%	89,26%	**93,39%**	92,00%
F1 score	70,26%	70,35%	75,31%	72,69%	78,61%	84,30%	87,54%	**91,96%**	90,79%

**Fig. 3:**
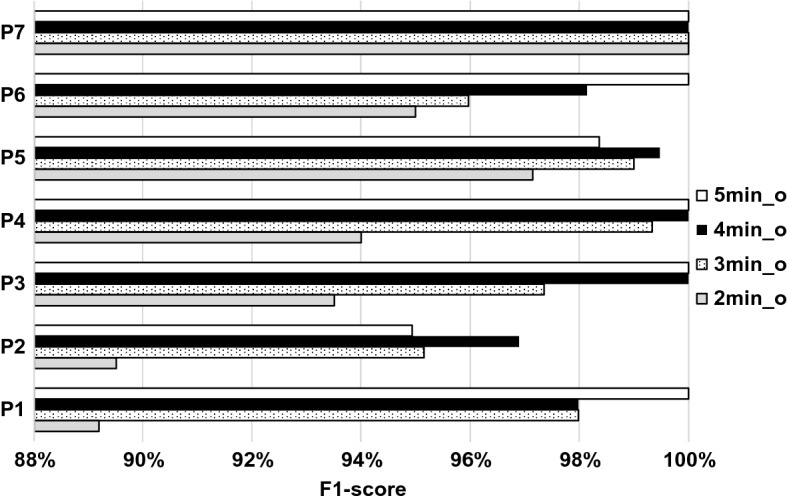
F1 scores for overlapping time windows, for participant-wise training models using all HRV features

The time window setups for the participant-wise modelling runs are shown in Table 6. The achieved performance is generally good, but individual scores vary. For example, all window setups yielded perfect detection results for P7, but even the best F1 score is below 97% for P2, while the majority of others’ scores are close or above 97%. A more detailed participant-wise overview of F1 scores for overlapping time window setups using all HRV features is shown in Fig. 3.

**Table 6:** The best-performing time window setups for the participant-wise training models

Participant	Figure of merit	All features		Time and non-linear features		Frequency features	
		Overlap	No overlap	Overlap	No overlap	Overlap	No overlap
P1	Window length	5 min	4 min	4, 5 min	5 min	5 min	4 min
	F1 score	100%	100%	100%	90.0%	97.5%	100%
P2	Window length	4 min	5 min	5 min	2 min	5 min	3 min
	F1 score	96.8%	92.1%	95.5%	90.7%	89.4%	92.4%
P3	Window length	4, 5 min	5 min	5 min	5 min	4 min	4 min
	F1 score	100%	100%	99.0%	100%	97.1%	94.6%
P4	Window length	4, 5 min	3 min	4, 5 min	5 min	2 min	4 min
	F1 score	100%	98.6%	100%	100%	96.7%	94.7%
P5	Window length	4 min	5 min	4, 5 min	5 min	3 min	5 min
	F1 score	99.5%	100%	100%	100%	96.4%	100%
P6	Window length	5 min	96.2%	3 min	5 min	5 min	4 min
	F1 score	100%	100%	98.0%	100%	95.6%	94.6%
P7	Window length	4, 5 min	all	1, 4, 5 min	5 min	4 min	5 min
	F1 score	100%	100%	100%	100%	100%	100%
Average	F1 score	99.47%	98.67%	98.93%	97.24%	96.10%	96.61%

**Fig. 4:**
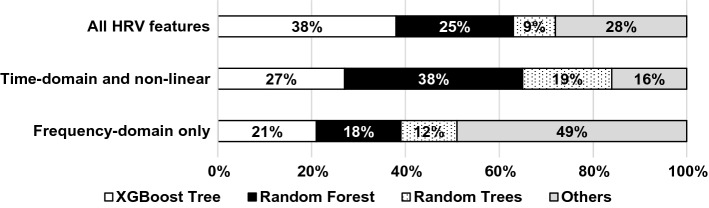
The classification algorithm-wise distribution of the best result achieved for all test runs in configuration 1, for each different feature set

The best-performing classification algorithms were the *XGBoost Tree*, the *Random Forest* and *Random Trees*. Figure 4 shows the distribution of the algorithms providing the best results regarding all model runs using configuration 1. *XGBoost Tree* performed best in most of the runs when all HRV features and frequency-domain features were used (38% of all runs) followed by *Random Forest* (25%) and *Random Trees* (9%). *Random Forest* was the most successful in the case of using time-domain and non-linear HRV features, followed by *XGBoost Tree* and *Random Trees*.

Model performance was more “balanced” in case of participant-wise classification, as there was no single model that could outperform the other ones in most cases. The top 6 best-performing algorithms in 65.79% of all cases were the *Random Trees* (11.59%), *XGBoost Tree* (11.36%), *Discriminant* (11.14%), *LSVM* (11.02%), *CHAID* (10.34%) and *Random Forest* (10.34%).

### Statistical results

The one-way analysis of variance (ANOVA) for configuration 1 has shown that significant differences in the model results were present for 6 of the 9 time window setups. The three setups with no significant differences were the 3, 4 and 5-min-long non-overlapping time windows. The t-tests have shown that the frequency-domain only features differed significantly from the other two feature sets for the overlapping window setups and the 2-min long non-overlapping setup. For the 1-min-long non-overlapping time window setup, the significant difference was between the time-domain and non-linear features set and the frequency-domain only set.

The statistical analysis of configuration 2 revealed that while model performance does vary with respect to the participant (as expected), the majority (78.7%) of these differences were not significant. 83.3% of the significant differences were attributed to participants P1 and P2. P1 was involved in 33.3%, P2 in 61.1% of these cases (mutually present 11.1%).

## Discussion

While the number of participants initially enrolled were comparable to some other research presented in the literature [10, 19, 26], the final count became rather low in our study due to the relatively high number of dropouts. Nevertheless, there are also precedents for having a similarly low number of participants [18, 23, 48] for stress detection purposes in small-scale studies. While a higher number of participants would allow a population level analysis of the natural variability of predictability, this was not the aim of the current study.

One main limitation of using HRV-based stress detection methods that must be considered is that their performance can drastically decrease for people with heart conditions causing arrhythmias (rhythm abnormalities), even to the point when they are not applicable. This is because HRV features are to be derived from regular/normal successive heartbeats. However, it must be noted that arrhythmias are not necessarily present constantly, and their presence can be negated with proper signal processing techniques in less serious conditions. While the number of cases is expected to grow in the following decades, the vast majority of the population is and will be unaffected. The most common heart rhythm disorder, atrial fibrillation, is estimated to have a prevalence of 3% in people aged 20 years or above [49] and a little higher for older adults (~ 4.84%) [50].

Based on the STAI scores, the saliva-cortisol test results, and some discussions with participants after the trial, the Trier Social Stress Test was indeed found to be quite effective in inducing stress in people aged 50 and above. The same cannot be said for the Stroop colour test, as no induced stress could be observed for most participants. Based on participant and investigator remarks, it seemed that for some, the fact that they had to use digital devices made the experience more like some sort of a game. They tended to enjoy the task rather than being stressed about having it completed. Meanwhile, less technologically proficient users seemed not interested in doing their best. Though using a digital version of the Stroop test requires less resources and evaluation is faster, these findings indicate that special care should be taken when choosing a stressor for older adults. Possible solutions could be making the digital version easier to use, finding methods for motivating participants more efficiently or including only people accustomed to using digital devices.

As the Stroop sessions’ ineffectiveness was noticed in time, incorrectly using those measurements as stressful samples could be avoided. While awakening intervals could be used instead to maintain a balanced stressful–non-stressful sample ratio, the possible differences between such “spontaneous” stress situations and provoked stress events such as the Trier test could be investigated further in a future study.

Another interesting topic related to methodology is using relaxation as a non-stressful period. There is no doubt that relaxation is not stressful, but one could argue that physiological features in everyday situations when no significant stress can be perceived are not the same as when individuals are relaxing. Therefore, high performing classifiers taught with only stressful and relaxing samples might prove less effective in everyday situations when the difference between stressful and non-stressful situations is smaller. Having measurements taken during neutral time periods, when participants are distracted with minor tasks (such as reading or small talk) instead of “doing nothing” might better simulate everyday non-stressful situations. Using such data could prove to provide better real-life classification performance, this is why neutral periods were used in our trial. Results showed that a limited time-domain/non-linear HRV feature set could achieve similar classification performance to that of all features, including frequency-domain. Thus, even with less computational resources, it is possible to adequately detect stress, supporting the assumption that low-cost AAL solutions could be used for such purposes. However, the performance of using only frequency-domain features was found to be just slightly lower (92.10% accuracy, 91.96% F1 score), meaning they could be an alternative if low-cost solutions explicitly designed for them are available.

The comparison of results for the different time window setups shows that classification performance improves with overlapping time windows. This is in line with previous research [19, 27], and follows form the fact that more data are generally expected to yield more precise estimations. Moreover, detecting the exact moment when changes are caused by stress can be more problematic with non-overlapping setups (especially for longer time windows). If, for example, the onset happens near the middle of the interval, the data recorded in the first part lower the level of change perceived for the entire window.

The best results were produced by using 5-min-long overlapping time windows. It might not seem an achievement compared to other studies where similar performances were achieved with shorter intervals (e.g., 50 or 60 s). However, relying on short intervals only is not a meaningful target as future portable devices are expected to facilitate ubiquitous monitoring techniques where users wearing the devices would not notice measurements being taken. No cooperation would be required, nor to have users interrupt their everyday activities. Smart bands and activity trackers already support this functionality at a certain level. It can be assumed that future advancements will make them achieve even more, supporting any preferred time window without any considerable limitations.

Moreover, using longer time windows could have additional benefits in real-life situations, as most results published are typically based on measurements taken in controlled environments. A system using shorter intervals is more likely to be affected by noise, such as sudden user movements or just the “usual” interferences related to using electronic devices. These effects can usually be negated more efficiently with longer time windows. Furthermore, while stress is known to have a “dynamic nature”, and there are indeed multiple cases for quick-onset stress situations (e.g., receiving devastating news or being frightened), acute stressors are not just like these. Some have a bit longer build-up period when frustration is constantly increasing up to a severe level (e.g., struggling with something or someone and getting annoyed), which could be missed by time windows that are too narrow. Such changes could be observed more easily using longer (but still short-time) time windows, without losing the ability to detect quick-onset events.

Concerning the general applicability of the models used, it can be concluded that significant differences between participants can occur even when adequate data are available (e.g., P2). This can be attributed to the natural physical variability present between different individuals, as some people react quite differently to the same impulses, while others’ reactions are easily predictable. However, it is important to note that even the results for participant P2 can still be considered quite good (89–97% F1 score).

### Comparison to related work

The results presented in this paper are similar to other ECG or PPG-based methods using HRV features and even better in some cases. In comparison with the results of Ham et al. [23], who have achieved 81–82% accuracy with non-overlapping 4-min-long time windows, we have achieved an accuracy of 86.67%, which could be increased to 94.60% by using overlapping time windows of the same length. Moridani et al. [20] reported an F1 score of 97.9% for differentiating between cognitive stress and relaxation using 5-min-long measurements. Our results for overlapping 5-min-long time windows using time-domain and non-linear HRV features were quite similar, with an F1 score of 96.26%.

**Fig. 5:**
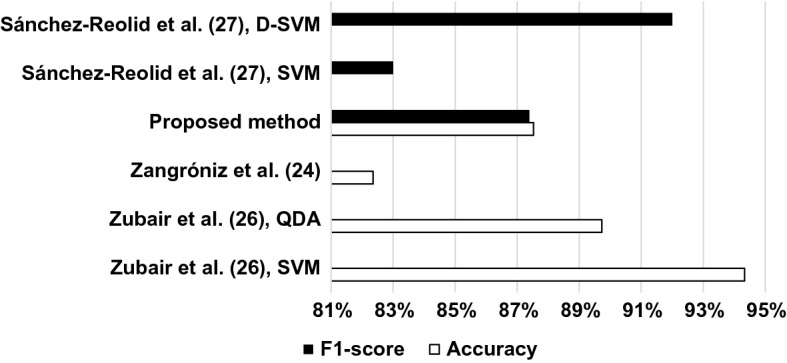
Performance comparison of methods using 60-s long, non-overlapping time windows

As shown in Fig. 5, if only methods based on similar window lengths (60 s) are compared, our results for time-domain and non-linear HRV feature sets (87.53% accuracy, 87.39% F1 score) are still better than that of Zangróniz et al. [24] (82,35% accuracy) and close to the QDA results (89.73% accuracy) of Zubair et al. [26] (but not as good as their SVM results with 94.33% accuracy), both using HRV features. The results obtained by Sánchez-Reolid et al. [27] with a different sensor (GSR) are similar to ours when SVM was used (83% F1 score), but their D-SVM solution is better (92% F1 score).

**Fig. 6:**
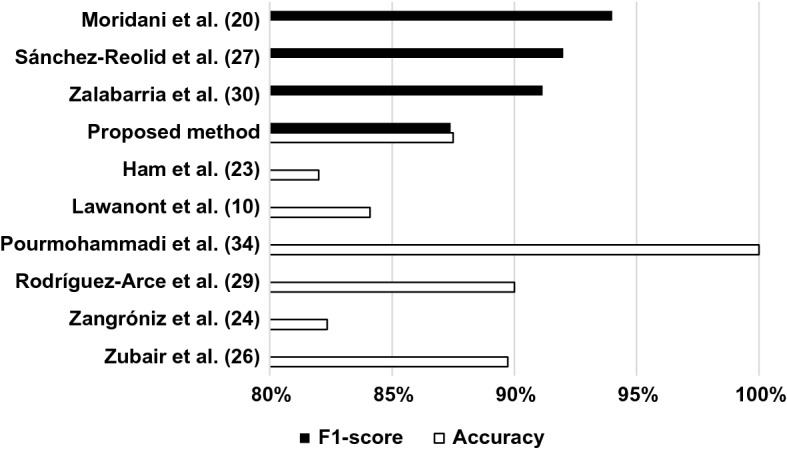
Performance comparison of best results achieved (different time window configurations)

The multimodal sensor solutions with shorter time windows presented by Rodríguez-Arce et al. [29] (90% accuracy) and Zalabarria et al. [30] (91.15% F1 score) also have better performance compared to our 60 s methods. As discussed previously, comparing results achieved with different time window lengths might not seem justifiable at first. However, already the 2-min-long overlapping windows for time-domain and non-linear HRV features are on par with these achievements with 91.77% accuracy and 91.62% F1 score. Furthermore, if the idea behind ideal AAL solutions is accepted, i.e. ubiquitous monitoring will be available in future AAL solutions, our best results achieved by 5-min overlapping time windows outperform most of the methods previously mentioned, with its 96% accuracy and F1 score, as shown in Fig. 6.

Only the 100% accuracy of Pourmohammadi et al. [34] using both EMG and ECG sensors and SVM could not be reached by models used in configuration 1. Their solution’s high performance could be partly attributed to their setup using the limb leads ECG configuration (one electrode on each hand and leg), instead of a portable sensor, which might have provided more accurate RR interval data to work with. While using the EMG solution described in their work might seem impractical first, future AAL devices such as the Vital Jacket used in [19] might provide a way for its everyday usage. It is certainly an interesting proposal that should be investigated further.

Configuration 2 results imply that relatively few validated recordings are needed to achieve high stress detection performance (90–100% F1 score) on an individual level. As expected, results indicate that individual differences (both physiological and psychological) cause prediction accuracy to be significantly different for each person. By testing different time window setups, it was possible to find which settings were the best for each participant, achieving high average classification performance (98.93% F1 score).

## Conclusion and future work

This study presented that effective stress detection for people aged 50 years or more is achievable with classification models using RR interval-based HRV data gathered via portable ECG sensors. The main result of the work is that the performance of the proposed prediction models matches those more complex solutions where multimodal measurements from various sensors were used, thus offering a less complex and expensive alternative for future AAL solutions. Moreover, it was also found that models based only on time-domain and non-linear HRV features could reach similar or even better performance (96.31% accuracy, 96.26% F1 score) than more computationally complex solutions including also frequency-domain features. A strength of the study is that it was performed with standardised and validated stressing methods, by testing multiple time window and input configurations, and using various classification algorithms to build detection models.

Preparation of a more detailed future trial is currently in progress at the time of writing this paper. The new experiment is planned to include more participants (about 50 people) from multiple age groups, to investigate the developed models’ performance by testing them on a broader population.

## Methods

### Study population

Data were gathered in a clinical study performed at the Cardiac Rehabilitation Institute of the Military Hospital, Balatonfüred, Hungary. The inclusion criteria were being aged 50 or above, having no previous history of cardiovascular conditions that would invalidate HRV measurements, and having no colour vision problems that would affect the execution of the Stroop test.

From the initial 12 participants who agreed to participate in the study, five had to be excluded. Two were excluded as they did not adhere to the study protocol. For two others, the ECG data recorded proved to be of low quality. Numerous extra heartbeats were found in one participant’s case, making the measurements unsuitable for HRV processing. The average age of the remaining seven participants (3 women, 4 men) was 63.14 years, with a standard deviation of 11.78. All of them were taking part in 3-week-long rehabilitation courses that consisted of daily activities similar to everyday life. All participants were under continuous medical and dietary supervision, and informed consents were obtained before their inclusion in the study.

The study protocol was prepared to comply with the World Medical Association Declaration of Helsinki on Ethical Principles for Medical Research Involving Human Subjects. Ethical approval was given by the National Institute of Pharmacy and Nutrition (OGYÉI), Budapest, Hungary, under submission number OGYÉI/4778/2018.

### Experimental protocol

Participants took part in two different stressing sessions, held on consecutive days, but during a similar time. For both sessions, first, the participants were escorted to a secluded and calm room where they filled out a copy of the Hungarian version of the STAI questionnaire [51]. A salivary cortisol test sample was also taken for the first batch of participants (i.e. the first 4 people). Then they were instructed to try to avoid negative and stressful thoughts while being seated and left alone for the next 10 min. After this resting phase, participants were escorted to a nearby room where the stressing began.

For the first session, participants performed the standardised Trier Social Stress Test. Participants were first informed about the details of the current session: two 5-min-long tasks had to be performed in front of a committee of 3 people (its member made up of individuals unknown by the participant), who were said to be behaviour experts analysing them. A camera and a microphone were also present in the room, said to be recording the interview for further analysis (they were not doing so). They had to complete the first task of making a speech as part of a job interview, ensuring the committee that they are the perfect candidate for the position, after an optional, at most 3-min-long preparation interval. As the second task, participants were asked to count down from 2023 by seventeens with as few mistakes as possible, by starting again whenever an error was made.

In the second stressing session, participants were seated at a table. They were given a tablet device to complete a version of the Stroop colour test. In 10 min, their task was to match colours to labels at an increasing pace and try and do as many correct matchings in a row as possible (i.e. getting the best “high score”). One additional point was given for each correct solution, and the score reset to 0 if a mistake was made.

After each stressing session, participants were escorted back to the starting room to fill out another copy of the STAI questionnaire. For the first batch, another salivary cortisol test sample was taken.

Besides taking part in the stressing sessions, participants were asked to keep a diary with notes on when they woke up or did notable physical activities (e.g., going for a walk, exercising). The diary wake-up times were validated by analysing the respective HRV recordings (for significant mean heart rate changes). Waking up in the morning is known to be a generally stressful situation as the body shifts from a resting-recovering state to an active-ready state. For participants where the awakening time could be validated this way, 10-min-long “awakening intervals” were extracted from their measurements to have additional stressful samples. With a similar methodology, some other time intervals that could be characterised as non-stressful were also selected for some participants to have the same amount of stressful and non-stressful measurements. These were usually taken from 30- to 60-min-long resting-like periods just before lunch at noon, when it could be validated that no physical or notable mental activities were done.

### Physiological measurements

The participants wore the portable Firstbeat Bodyguard 2 ECG sensor [8], a low-cost AAL device providing RR interval measurements. The device operates as a one-channel ECG, i.e. by using two electrodes (one placed on the right side of the body under the collarbone, the other on the left side of the body on the rib cage), with a sampling frequency of 1000 Hz (with 1 ms precision). Participants were asked to wear the device for at least 2–2.5 consecutive days (except when showering/bathing), starting from the night before the first stressing session until the morning after the second session.

The RR interval data recorded by the sensors was pre-processed with Kubios HRV Standard software (version 3.3.1), with its threshold-based beat correction algorithm to identify and remove possible artefacts [52]. “Low” threshold (of value 0.3) was selected based on the literature [53] in order to provide a method that could be expected to work well with younger adults too. Kubios was also used to calculate the HRV features from the RR intervals.

Previous works have shown that using multiple different window length configurations can influence stress detection capabilities [19, 27]. Therefore, the classification algorithms were tested with 1-min (ultra-short), 2, 3, 4 and 5-min (short) window lengths. Moreover, both overlapping and non-overlapping configurations were tested for each interval. For overlapping configurations, the subsequent time windows started 1 min after the previous window’s start. Table 7 shows the total data amount used for each participant.

**Table 7:** The amount of data used for training and testing purposes for each participant

Total length of	P1 (min)	P2 (min)	P3 (min)	P4 (min)	P5 (min)	P6 (min)	P7 (min)
Stressful records	20	50	40	27	44	37	20
Non-stressful records	20	50	40	27	44	37	20

Only for the first four participants was it possible to use saliva-cortisol tests right before and after each of the stressing sessions due to logistic reasons. The samples were taken by medical personnel and were immediately transported to the scientific laboratory for analysis.

### Heart rate variability features

Kubios can calculate 52 features from source data if the covered time interval contains enough measurements for the calculation. 13 of them are time-domain features, 7 are non-linear, and 16–16 frequency-domain features are calculated by both Fast Fourier transformation (FFT) and parametric autoregressive (AR) modelling (called FFT and AR spectrum results), respectively.

Amongst the time-domain features are:

- the means and standard deviations for the RR intervals and the heart rate;
- the root mean square of the successive differences (RMSSD);
- the RR tri-index;
- the triangular interpolation of RR intervals (TINN);
- the number of successive RR intervals that differ more than xx milliseconds (NNxx), and the ratio of NNxx and the total number of RR intervals (pNNxx). During the trial, the default value of 50 ms was used for xx.

Frequency-domain features include:

- the very low frequency (VLF), low frequency (LF) and high frequency (HF) components for the peak frequencies (Hz), and the absolute (ms^2^ and log) and relative (%) powers;
- the LF/HF ratio;
- the total power (ms^2^) and the normalised (n.u.) powers for LF and HF.

The non-linear features are:

- the metrics used for the Poincare-plot (SD1, SD2, SD2/SD1);
- the approximate and sample entropies;
- the alpha 1 and 2 values of the detrended fluctuation analysis (DFA).

More information about the exact HRV features is available at [54].

### Classifier models, model training

In order to investigate multiple different classification algorithms and methods, SPSS Modeller 18.2.1 was used. A total of 15 different classifier types were used in two different configurations: C&R Tree (Classification and Regression), C5, CHAID (Chi-square Automatic Interaction Detector), Decision List, Discriminant, Logistic regression, LSVM (linear support vector machine), Neural Net, Quest, Random Forest, Random Trees, SVM (support vector machine), Tree-AS, XGBoost Linear and XGBoost Tree. Further details can be found in [55].

In configuration 1, the available features were used to form three feature sets: one containing all available features, one for the time-domain and non-linear features, and one for the frequency-domain features only. The rationale behind this is that calculating frequency features is generally considered more computationally complex and resource-intensive than time-domain and non-linear features. If models’ performance using all other features does not differ significantly from those using frequency-domain features, they could provide a more effective method for stress detection. Performance with frequency-domain features only was also investigated to see if solutions explicitly designed for frequency-domain computations could be beneficial.

The model training process was executed by using 2/3 (67%) of the available records for the training set and the remaining 1/3 (33%) for the testing set (2:1 ratio). Records were randomly sampled into these two sets for each run, by using the built-in sample nodes of the SPSS modeller. Sampling and training were executed ten times for each of the different model configurations tested.

In configuration 2, the training and testing sets were built individually for each participant, without using data from other participants. For this purpose, each participant’s stressful and non-stressful records were randomly sampled one-by-one into the participant-specific training and testing sets, maintaining a 2:1 testing–training ratio. As in configuration 1, sampling and model building was repeated ten times for everyone, and the performance of the three different feature sets (all, time and non-linear, frequency) was compared.

### Performance metrics and statistics

Solutions given by classifier models were categorised into four result type groups. The correctly categorised ones into true positives (*TP*) and true negatives (*TN*), while the incorrect ones into false positives (*FP*) or false negatives (*FN*). The following four metrics were used to evaluate classifier performance:

Accuracy: the ratio of correctly classified items and all items:

1 $$Acc= \frac{TP+TN}{TP+TN+FP+FN}.$$

Specificity: the ratio of correctly classified non-stressful items and all non-stressful items:

2 $$Sp= \frac{TN}{TN+FP}.$$

Sensitivity: the ratio of correctly classified stressful items and all stressful items (also known as recall):

3 $$Se= \frac{TP}{TP+FN}.$$

*F1 score*: a generally accepted field of merit for binary predictors, defined as the harmonic mean of precision ( $TP/(TP+FP)$) and recall:

4 $$F1= \frac{2\cdot TP}{2\cdot TP+FN+FP}.$$

The performance metrics listed above were calculated for all configurations in each run, using the classification algorithm provided by the best model, i.e. the values discussed in “Results” for the above configurations are each an average of 10 modelling runs.

The performance of the various classification algorithms was evaluated according to a marking scheme. The mark was the number of times the algorithm provided the best accuracy amongst all candidate algorithms and the accuracy was 85% or above, in order to avoid rewarding relatively good but still poor results.

To compare the results obtained for the different feature sets (all parameters, time-domain and non-linear, frequency-domain), a one-way analysis of variance (ANOVA) was performed to identify if statistically significant differences could be found (with p < 0.05). If a significant difference could be observed, Student’s t-test was used to find which feature sets were different. These techniques were also used to check significant differences amongst participant-wise model results.

## Supplementary Information

**Additional file 1.** The measurement data (RR intervals) used in model building.

## Data Availability

The detailed, anonymised data sets used are available at request from the corresponding author and as a supplement to this article. Other data related to the clinical trial may be released upon application to the institutional Ethical Committee of the Military Hospital, which can be contacted at Magyar Honvédség Egészségügyi Központ Intézményi és Regionális Kutatásetikai Bizottsága, Róbert Károly körút 44, 1134 Budapest, Hungary.

## Abbreviations

AAL: Ambient Assisted Living
ANOVA: Analysis of variance
DFA: Detrended fluctuation analysis
ECG: Electrocardiogram
EDA: Electrodermal activity
EMG: Electromyography
GSR: Galvanic skin response
HRV: Heart rate variability
PPG: Photoplethysmogram
RR (interval): The time interval between R peaks of successive QRS complexes
STAI: State–Trait Anxiety Inventory
(D-)SVM: (Deep) Support Vector Machine
VLF, LF, HF: Very low frequency, low frequency, high frequency
